# Dentition as One of the Causes of Cholera Infantum

**Published:** 1878-11

**Authors:** J. H. Nowlin

**Affiliations:** Little Rock, Ark.


### ARTICLE IV.

### *Is Dentition one of the Causes of Cholera Infantum? If So, In What Way.*

BY J. H. NOWLIN, M. D., LITTLE ROCK, ARK.

In your issue of June 15th I read, with interest, an article from the pen of William S. Stewart, A. M., M. D., on "Some Considerations in regard to the Causes of Cholera Infantum." After mentioning several causes, and giving his reason for considering them such, he continues as follows—"*Teething* may be considered a concomitant cause of cholera infantum, as *it is during* dentition (all italics mine) that it occurs; undoubtedly the one is a great aggravation of the other. To what extent, if at all, dentition may be a *cause* of this disease, authors seem to take little interest," etc. And again—"It has been in *theory*, to me, one of the *chief* causes of the disease." Unfortunately for his readers, the Doctor failed to give specifically and definitely his *theory* upon the subject. It would have been much more satisfactory than to have dismissed the matter as he has done, with the statement of the simple facts that "the disease occurs during dentition." That "we have as one of the first symptoms an excitement of the *buccal* and *salivary* glands of the *mouth*," "their secretions being more (than usually) acid," etc. Now, it is said, "Doctors will differ;" indeed, the saying long since passed into a proverb; but why, I am not prepared to say, as men of all other professions and persuasions differ quite as widely as doctors. For example, lawyers, clergymen, etc., than whom none can differ more.

When a student, nearly four decades in the past, I was taught to believe that cholera infantum often occurred during the first few weeks, or even days, of the earthly existence of children, long before the "period of dentition," or even the "excitement of the buccal and salivary glands of the mouth." My experience, thus far, has fully confirmed the teaching of my preceptors in this particular. I was also taught, and pretty fully imbibed the view, that dentition, or the process of teething, was as really and truly a physiological one, as the growth and development to perfection of any other part of the animal economy. Nor have I ever yet had any good reason to believe otherwise than that it is commenced and perfected with as little suffering or detriment to the young animal as the growth of any other part of its being. Moreover, I am not aware that any professional man ever maintained the contrary, except (miserabile dictu !) in relation to the genus homo.

Why this exception ? The process is precisely the same in the brute as in the human, in utero, as during the first, second, third, or fifth year after birth. What is there in the growth of the teeth, and the absorption of the gums, to give place to the former (the whole of the process,) that can possibly produce irritation in any degree, not to say sufficient to give origin, by sympathy (reflex action,) or continuity of surface, to laryngitis, croup, bronchitis, or the various troubles of the alimentary tube ? Is absorption an inflammatory instead of a physiological act ? I have never so considered it. Where, then, is the seat, and what is the morbid action thought to be so potential of mischief ? So far I have searched for it in vain. Can it be in the failure of the absorbents to remove the very delicate and vascular gum tissue, containing such an abundance of nerve filaments, so that the unyielding and persistently progressing teeth impinge upon the former with such force as to bring into existence, through this nerve element, the terrible consequences so frightful to contemplate, which we see in the journals of the present day, as well as those of past ages.

If this should be regarded the true solution of the difficulty, I would ask why the morbid phenomena are postponed, for the most part, till only a very short time before the eruption of the teeth? If absorption is wanting entirely, pressure must begin with the growth of the teeth, and be exerted, not only upward, but around their entire circumference. The resulting evils would then be seen from birth to the close of the dentition period, but diminishing as the teeth approach the surface of the gums, the resistance becoming less and less till the teeth are liberated. With much interest, and for many years, I have noted and canvassed the views expressed and defended by the representative men of our profession, and have seen, with pleasure, a gradual but steady falling off of the advocates of the old theories of the multiform evils of dentition. Indeed, at this moment, I cannot mention one on either continent, who really deserves to be considered in the upper story of our noble profession, who still adheres to the views of John Hunter, Marshall Hall, etc., great as those men were and are deservedly considered.

During the early part of my professional labors, say through the fifth and half of the sixth decades of this century, in conformity to the customs of the time, as well as in following (in a small way) the precepts and examples of the eminent John Hunter, who said he "was not ashamed to acknowledge that he had cut the gums over the same teeth as often as ten times;" and of Marshall Hall, who said to his class, "In a difficult case I would not hesitate to recommend the incision of the gums *every* day, and even twice a day;" beside many other very eminent men since their day, I occasionally was induced to split the gums and give the teeth a chance to escape from their imprisonment, and the infant from its perilous condition. Close observation, however, followed assiduously, failed, in a single instance, to reveal any good result, unmistakably traceable to the operation. I always cut down to the tooth, as evinced by the grating of the knife upon its crown; yet I never saw a tooth, when thus freed, jump through the incision, as might have been expected from the amount of pressure supposed to be exerted upon it by the resisting gums! Another very extraordinary observation I made, quite in conflict with the pressure theory, viz., that no such thing ever occurred as retraction of the edges of the cut, although the tooth might be seen by the naked eye if the cut edges were forced asunder; and, besides, if the tooth was not too nearly through to permit adhesion, the opposing edges would grow together in a few hours, mauger the inflammation, swelling, pressure, etc., supposed to be present.

These observations, with others of like character, convinced me long ago that the world had been most immensely, detrimentally, and unmercifully humbugged by our profession, upon the subject of "first dentition;" and hence, for the last ten years, at least, I have fought earnestly and most persistently against the teachings of a majority of the profession, who still adhere to the old theories, as well as against the current and popular views upon the subject, as quite a number of my professional brethren can testify. Mothers I sometimes have found it difficult to convince. Wedded to the belief imbibed from their parents or family physicians, who knew all that could be learned, in their opinion, they have, or seem to have, no desire to know anything to the contrary. A mother presents a child eight to eighteen months old, and wishes me to examine its gums. Its bowels are "running off;" it "has little appetite," "has lost flesh, and is very fretful," etc. I reply, "Now, my good madam, put your finger upon the tender gum, and see if the child suffers from the pressure." "O, no sir!" she immediately replies, "the child loves to have me press upon its gums, and it loves to bite on the rubber ring and any hard substance it can get hold of." "Well, madam, do you not suppose your child's gums are as tender and as easily hurt as your own." "Of course I do, sir." "Very well; now if your gums were inflamed, swelled, tender and painful, do you believe you could bite the rubber ring and other hard substances, and permit your gums to be pressed upon, like your child does." This is coming to rather close quarters for the good woman, and she makes a flank movement by saying, " O, sir, I think the baby's gums must itch him very badly ! "

I feel, Mr. Editor, that it is full time our profession had begun to undo some things which they have long done, and to unteach many things they have taught, which have given origin to much of the empiricism of the day.—*Med. and Surg. Reporter.*

